# Profound reduction in tamoxifen active metabolite endoxifen in a breast cancer patient treated with rifampin prior to initiation of an anti-TNFα biologic for ulcerative colitis: a case report

**DOI:** 10.1186/s12885-016-2342-x

**Published:** 2016-05-11

**Authors:** Sara L. Henderson, Wendy A. Teft, Richard B. Kim

**Affiliations:** Division of Clinical Pharmacology, Department of Medicine, 339 Windermere Road B9-130, London, ON N6A 5A5 Canada; Pharmacy Services London Health Sciences Centre, Western University, London, ON Canada

**Keywords:** Tamoxifen metabolism, Rifampin, Endoxifen, CYP2D6, Drug-drug interaction

## Abstract

**Background:**

Tamoxifen, a common anti-estrogen breast cancer medication, is a prodrug that undergoes bioactivation *via* cytochrome P450 enzymes, CYP2D6 and to a lesser degree, CYP3A4 to form the active metabolite endoxifen. With an increasing use of oral anti-cancer drugs, the risk for drug-drug interactions mediated by enzyme inhibitors and inducers may also be expected to increase. Here we report the first case demonstrating a potent drug-drug interaction in a real-world clinical setting between tamoxifen and rifampin in a breast cancer patient being treated concurrently for ulcerative colitis.

**Case presentation:**

We describe a patient on adjuvant tamoxifen therapy for breast cancer that was prescribed rifampin for TB prophylaxis prior to initiation of an anti-tumor necrosis factor (TNF)-α agent due to worsening ulcerative colitis. This 39 year old Caucasian woman had been followed by our personalized medicine clinic where CYP2D6 genotyping and therapeutic monitoring of tamoxifen and endoxifen levels had been carried out. The patient, known to be a CYP2D6 intermediate metabolizer, had a previous history of therapeutic endoxifen levels. Upon admission to hospital for a major flare of her ulcerative colitis a clinical decision was made to initiate an anti-TNFα biological agent. Due to concerns regarding latent TB, rifampin as an anti-mycobacterial agent was initiated which the patient was only able tolerate for 10 days. Interestingly, her plasma endoxifen concentration measured 2 weeks after cessation of rifampin was sub-therapeutic at 15.8 nM and well below her previous endoxifen levels which exceeded 40 nM.

**Conclusion:**

Rifampin should be avoided in patients on tamoxifen therapy for breast cancer unless continued tamoxifen efficacy can be assured through endoxifen monitoring. Drug-drug interactions can pose a significant risk of sub-therapeutic benefit in tamoxifen patients.

**Electronic supplementary material:**

The online version of this article (doi:10.1186/s12885-016-2342-x) contains supplementary material, which is available to authorized users.

## Background

Tamoxifen is a selective estrogen receptor modulator that is indicated as first-line and adjuvant treatment in estrogen-receptor positive breast cancer. It has been shown to decrease the risk of recurrence of breast cancer, as well as the risk of mortality [1]. Tamoxifen is a prodrug that is extensively metabolized in the liver. The efficacy of this drug is related to achieving therapeutic plasma levels of both tamoxifen and its active metabolite 4-hydroxy-N-desmethyl-tamoxifen, otherwise known as endoxifen [2]. Tamoxifen is converted to primary metabolites N-desmethyl-tamoxifen (NDM-tam) and 4-hydroxy-tamoxifen (4-OH-tam) which are both further converted to endoxifen mainly by the cytochrome P450 (CYP) enzyme CYP2D6 and to a lesser extent by CYP3A4 [3]. It has been well established that patients with CYP2D6 genetic deficiencies have lower endoxifen levels which may place these patients at risk for being within a sub-therapeutic range [4, 5]. Given the increasing use of oral anticancer medications, like tamoxifen [6], the risk for drug-drug interactions (DDI) mediated by enzyme inhibitors and inducers may also be expected to increase. Our group has recently reported a DDI between the enzyme inducer phenytoin and tamoxifen which resulted in profound reduction of endoxifen levels [7], indicating that DDIs in addition to genetic deficiencies pose a real-world risk for tamoxifen patients.

As CYP3A4 plays a role in converting tamoxifen to endoxifen, it was thought that CYP3A4 induction by drugs such as rifampin may result in increased endoxifen levels, thus mitigating the low levels often seen among patients with CYP2D6 deficiencies. A recent pharmacokinetic study designed to test this hypothesis was prematurely stopped when the first four subjects had a marked decrease in endoxifen levels after rifampin administration [8]. Here, we present to our knowledge the first case report demonstrating a potent DDI in a real-world clinical setting where a patient was being treated concurrently with rifampin while on tamoxifen therapy. While oral anticancer agents are likely associated with improved quality of life and convenient for oncology patients [9], clinically relevant DDIs may have a significant impact on the efficacy of tamoxifen treatment.

## Case presentation

### Relevant medical history

**Fig. 1:**
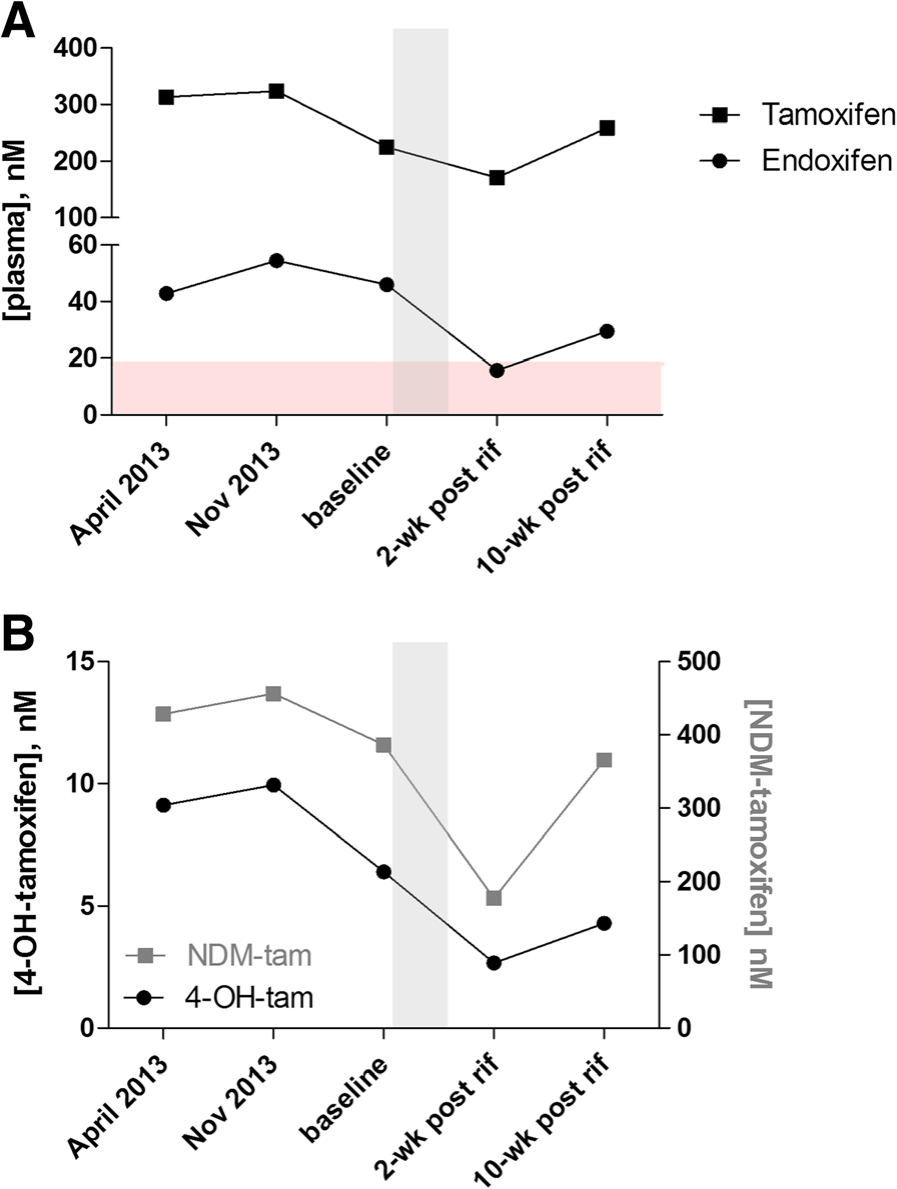
Plasma levels of tamoxifen and metabolites before and after rifampin administration. Blood samples were obtained prior to rifampin treatment with baseline collected right before rifampin initiation. Follow-up samples were collected 2 and 10 weeks post-rifampin discontinuation. Plasma concentrations of tamoxifen and endoxifen (**a**) and primary metabolites, NDM-tamoxifen and 4-OH-tamoxifen (**b**) were measured from each blood sample. Horizontal shaded region in (**a**) depicts the sub-therapeutic range. Vertical shaded region in (**a**) and (**b**) depicts the time period of rifampin treatment

In April 2013, a 38-year-old woman was referred to our Personalized Medicine Tamoxifen Clinic for assessment. One year previously she had been diagnosed with right-breast invasive carcinoma, stage T1cN0M0, estrogen/progesterone receptor positive, human epidermal growth factor receptor 2/neu negative, and underwent right segmental mastectomy and subsequent radiation. In January 2013, she was started on adjuvant tamoxifen at a dose of 20 mg daily. The patient provided written informed consent for participation in our approved study allowing for pharmacogenetic testing of CYP2D6 and drug level analysis of tamoxifen and endoxifen levels using an established liquid chromatography-tandem mass spectrometry method [5]. Other medical history included a 12-year history of ulcerative colitis and occasional migraine headaches. In addition to tamoxifen, her medications at this time included ascorbic acid, ferrous gluconate, and 5-aminosalicylic acid. Results of CYP2D6 genotyping revealed a genotype of *1/*4, indicating that she was an intermediate metabolizer. The tamoxifen level of the patient was 313.29 nM and the endoxifen level was 42.89 nM, considered to be well within therapeutic range (Fig. 1a, Table 1, Additional file 1). Although she did report complaints of hot flashes and moodiness, she was otherwise able to tolerate tamoxifen. A follow-up visit in November 2013 to our clinic again revealed tamoxifen and endoxifen levels within therapeutic range.

**Table 1:** Drug levels and metabolite/parent ratios before and after rifampin administration

	Blood sample		
	Baseline	2-weeks post rifampin	10-weeks post rifampin
Analyte, nM (%)^a^			
Tamoxifen	225.25	171.18 (24.0)	259.12 (0.0)
NDM-tam	386.32	177.38 (54.1)	365.66 (5.4)
4-OH-tam	6.41	2.67 (58.3)	4.29 (33.0)
Endoxifen	46.03	15.75 (65.8)	29.45 (36.0)
Metabolite/Parent ratio (%)^a^			
NDM-tam/tamoxifen	1.715	1.036 (39.6)	1.411 (17.8)
4-OH-tam/tamoxifen	0.028	0.016 (45.1)	0.017 (41.8)
endoxifen/tamoxifen	0.204	0.092 (55.0)	0.114 (44.4)
endoxifen/NDM-tam	0.119	0.089 (25.5)	0.081 (32.4)
endoxifen/4-OH-tam	7.185	5.892 (18.0)	6.862 (4.5)

^a^% reduction from baseline measurement

### Clinical consult for ulcerative colitis flare

In July 2014, this patient suffered a flare of her ulcerative colitis and was admitted to hospital for management. The patient tested positive for latent tuberculosis during TB skin testing routinely performed prior to anti-TNFα monoclonal antibody treatment. As a result, she was to be started on 4 months of preventative tuberculosis therapy with rifampin. Our team was then asked to see her for assessment of a potential drug interaction between rifampin and tamoxifen. Other medications at this time included ascorbic acid, vitamin D, dalteparin, ferrous gluconate, and methylprednisolone. Repeat baseline tamoxifen and endoxifen levels were measured, and were consistent with previous testing (tamoxifen 225.25 nM, endoxifen 46.03 nM). Upon initiation of rifampin we anticipated a reduction of endoxifen due to the previous clinical study by Binkhorst et al, but predicted this patient would remain in therapeutic range based on her baseline endoxifen levels. She was discharged several days later with a prescription for rifampin 600 mg daily for 4 months but was only able to tolerate rifampin for 10 days. She was then re-admitted to hospital due to worsening ulcerative colitis and the rifampin was discontinued. Two weeks later, we obtained a blood sample for repeat tamoxifen and endoxifen levels. The tamoxifen level was 171.18 nM, approximately 24 % lower than baseline; while the endoxifen level was reduced by 66 % to 15.75 nM (Fig. 1a). The decreased level of endoxifen was not due to the lower tamoxifen level as the endoxifen/tamoxifen ratio was 55 % lower following rifampin treatment (0.09) compared to the baseline ratio (0.2) (Table 1). At 10 weeks post discontinuation of rifampin, measured endoxifen level had nearly doubled at 29.45 nM (Fig. 1a, Table 1), placing the patient back within therapeutic range.

In addition to tamoxifen and endoxifen levels, we measured primary metabolites NDM-tam and 4-OH-tam from each blood sample (Fig. 1b). We observed a greater than 50 % reduction of NDM-tam and 4-OH tam levels following rifampin treatment. A similar rebound effect of primary metabolites was observed at 10 weeks post-discontinuation of rifampin. While a reduction in the metabolite/parent ratios was observed at each conversion step in the tamoxifen to endoxifen pathway (Table 1), the most significant ratio reduction was noted for NDM-tam/tamoxifen and 4-OH-tam/tamoxifen (40 and 45 % reduction, respectively) suggesting the conversion of tamoxifen to either primary metabolite was rate limiting to the formation of endoxifen (Table 1).

## Discussion

Tamoxifen undergoes oxidative biotransformation to its metabolites primarily by the CYP enzymes CYP3A4 and CYP2D6. In particular, endoxifen formation, the main active metabolite thought to be responsible for tamoxifen’s therapeutic effect, is catalyzed by the CYP2D6 enzyme [4, 10]. It was therefore traditionally hypothesized that the concurrent use of potent CYP3A4 inducers, such as rifampin, may increase the amount of endoxifen formed, and in fact have a beneficial effect on the clinical outcomes of tamoxifen users, particularly among CYP2D6 poor metabolizers (PM). The effect of potent inducers such as rifampin can be multifactorial. We have shown that nuclear receptors such as Pregnane X Receptor (PXR) and Hepatocyte Nuclear Receptor 4α (HNF4α) regulate CYP3A4 expression [11]. Therefore, in principle, activation of PXR would be predicted to increase the level of endoxifen. However, we now know that endoxifen clearance may be further enhanced through the action of phase II enzymes as well as drug transporters. Indeed, our group has shown that endoxifen is an excellent substrate for P-glycoprotein and that central nervous system entry of endoxifen was nearly 20-fold greater in P-glycoprotein deficient mice, due to its absence at the blood brain barrier [12]. P-glycoprotein is also highly expressed in the apical domain of enterocytes thus reducing substrate drug absorption, while its expression on the canalicular domain of hepatocytes facilitates biliary excretion [13]. In addition, induction of glucuronidation is also likely involved in the enhanced clearance of tamoxifen and its metabolites [14]. Rifampin has been shown to induce several conjugating enzymes, including the uridine 5’-diphospho-glucuronosyltransferase (UGT) enzyme that catalyzes glucuronidation [15]. An interaction between rifampin and tamoxifen was noted in the late 1990s when a study showed a marked reduction in tamoxifen levels [16]. However, in terms of clinical impact, only recently have reports demonstrated drugs such as phenytoin and rifampin may have a deleterious effect on endoxifen formation [7, 8]. Based on the previous hypothesis that concurrent use of rifampin and tamoxifen would enhance endoxifen levels, the clinical trial published by Binkhorst et al was designed to show a beneficial effect among breast cancer patients on tamoxifen therapy [8]. However, the investigators terminated the study prematurely due to interim analysis data that showed profound reduction in endoxifen levels.

**Fig. 2:**
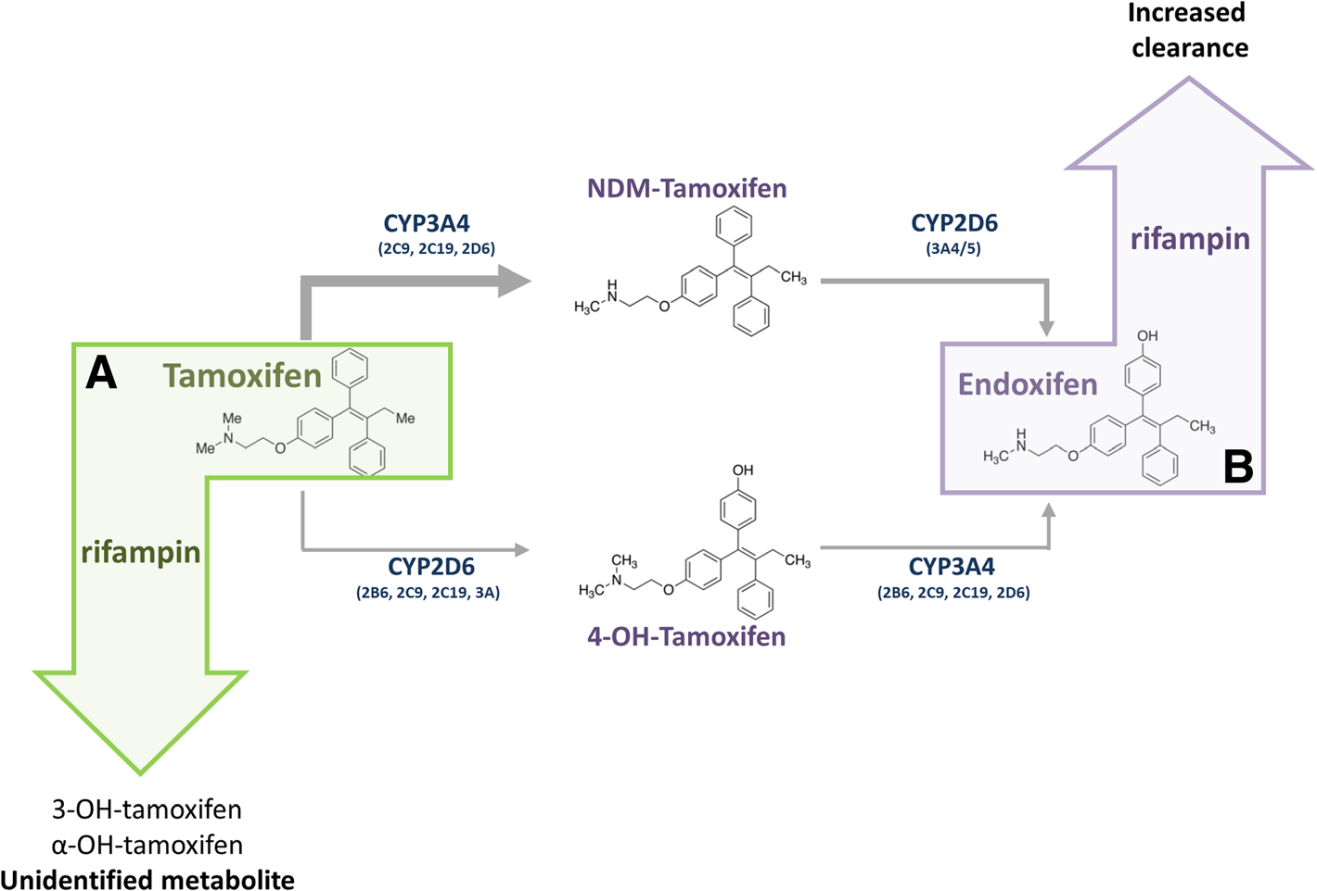
Proposed pathways induced by rifampin resulting in decreased endoxifen levels. Tamoxifen is bioactivated by CYP enzymes to form primary metabolites, NDM-tamoxifen and 4-OH-tamoxifen which are both further converted to the active metabolite endoxifen. Rifampin may lead to the marked reduction in endoxifen levels by (**a**) shunting tamoxifen metabolism to form alternate metabolites or (**b**) inducing phase II conjugating enzymes or drug transporters, such as P-glycoprotein, resulting in increased clearance of endoxifen

Here, we had the opportunity to investigate a potential DDI in a patient requiring both tamoxifen and rifampin. Through measurement of tamoxifen and metabolite levels prior to and following rifampin administration we were able to clearly document the reduction in metabolite formation. As the bioactivation of tamoxifen is complex, involving multiple drug metabolizing enzymes and transporters for metabolite clearance, we suggest that rifampin induction may play a role in multiple, non-mutually exclusive pathways resulting in lower systemic exposure of endoxifen. Induction of phase II enzymes and P-glycoprotein may result in increased clearance of endoxifen (Fig. 2). Additionally, the large reduction in the ratio of either primary metabolite to tamoxifen suggests that tamoxifen may in fact be metabolized to an alternate metabolite, reducing the formation of NDM-tam and 4-OH-tam, which are necessary for endoxifen formation (Fig. 2). It is important to note that the absorption and bioavailability of many drugs may be affected in the setting of inflammatory bowel disease. However, here we were able to measure the ratio of endoxifen to tamoxifen at several time points during the UC flare, including just prior to rifampin initiation (considered as baseline) and two weeks post rifampin discontinuation. The profound reduction in the endoxifen/tamoxifen ratio following rifampin treatment suggests that endoxifen is being cleared more rapidly than at baseline. Therefore, although we can’t rule out altered gut absorption, we predict this effect is primarily due to the drug interaction and not the disease setting.

Rifampin is used in the treatment of a number of infections, including those caused by tuberculous or non-tuberculous mycobacterium, and methicillin-susceptible or methicillin-resistant staphylococci. This broad spectrum of coverage likely means that its use remains consistent, particularly in the setting of a large, tertiary hospital site. Additionally, the use of tamoxifen is widespread in those who have received a diagnosis of breast cancer, as well as for specific populations of women at high-risk for cancer. Therefore, it is likely that a significant number of patients may be receiving concurrent therapy with both agents. We are struck by the magnitude of the inductive effect of rifampin on tamoxifen metabolism. A near 3-fold reduction in endoxifen level after only 10 days of rifampin therapy would suggest most patients on such combinations would be predicted to lose therapeutic benefit from tamoxifen placing them at a higher risk of recurrence.

## Conclusion

To our knowledge, this is the first case report in a real-world clinical setting that documents the profound effect of rifampin on endoxifen level. Importantly, we provide tamoxifen and endoxifen levels before and after rifampin therapy that clearly demonstrate the deleterious effect of rifampin to tamoxifen therapy. We believe that patients taking both tamoxifen and rifampin are at an increased risk for breast cancer recurrence or incidence, which may be of particular concern among high-risk patients, and should be avoided when possible. If it is essential that both agents be used, this case also highlights the importance of therapeutic drug monitoring of tamoxifen and endoxifen to optimize therapy.

### Ethics consent

Written informed consent was obtained from the patient for measuring drug levels and pharmacogenetic testing as approved by the Research Ethics Board, the University of Western Ontario (REB 15586).

### Consent to publish

Written informed consent was obtained from the patient for publication of this Case report. A copy of the written consent is available for review by the Editor of this journal.

### Availability of data and materials

The datasets supporting the conclusions of this article are included within the article (and its additional files).

## Additional file

Additional file 1: Spreadsheet of analyte concentrations and metabolite/parent ratios for tamoxifen and its metabolites. (XLSX 10 kb)

## Abbreviations

4-OH-tam: 4-hydroxy-tamoxifen
CYP: cytochrome P450 enzymes
DDI: drug-drug interaction
HNF4α: hepatocyte nuclear receptor 4-alpha
NDM-tam: N-desmethyl-tamoxifen
PXR: pregnane X receptor
TNFα: tumor necrosis factor alpha
UGT: uridine 5’-diphospho-glucuronosyltransferase
